# Maternal Control of PIN1 Is Required for Female Gametophyte Development in Arabidopsis

**DOI:** 10.1371/journal.pone.0066148

**Published:** 2013-06-17

**Authors:** Luca Ceccato, Simona Masiero, Dola Sinha Roy, Stefano Bencivenga, Irma Roig-Villanova, Franck Anicet Ditengou, Klaus Palme, Rüdiger Simon, Lucia Colombo

**Affiliations:** 1 Dipartimento di BioScienze, Università degli Studi di Milano, Milano, Italy; 2 Institute of Biology II, Faculty of Biology, Albert-Ludwigs-Universität of Freiburg, Schänzlestrasse 1, Freiburg, Germany; 3 Institut für Entwicklungsgenetik, Heinrich-Heine Universität, Düsseldorf, Germany; 4 Istituto di Biofisica, Consiglio Nazionale delle Ricerche, Milano, Italy; Umeå Plant Science Centre, Sweden

## Abstract

Land plants are characterised by haplo-diploid life cycles, and developing ovules are the organs in which the haploid and diploid generations coexist. Recently it has been shown that hormones such as auxin and cytokinins play important roles in ovule development and patterning. The establishment and regulation of auxin levels in cells is predominantly determined by the activity of the auxin efflux carrier proteins PIN-FORMED (PIN). To study the roles of *PIN1* and *PIN3* during ovule development we have used mutant alleles of both genes and also perturbed *PIN1* and *PIN3* expression using micro-RNAs controlled by the ovule specific *DEFH9 (DEFIFICENS Homologue 9)* promoter. *PIN1* down-regulation and *pin1-5* mutation severely affect female gametophyte development since embryo sacs arrest at the mono- and/or bi-nuclear stages (FG1 and FG3 stage). PIN3 function is not required for ovule development in wild-type or *PIN1*-silenced plants. We show that sporophytically expressed *PIN1* is required for megagametogenesis, suggesting that sporophytic auxin flux might control the early stages of female gametophyte development, although auxin response is not visible in developing embryo sacs.

## Introduction

In flowering plants, the gametophytes are comprised of only a few cells embedded within the diploid sexual organs of the flower. The formation of the female gametophyte is divided into two main steps, megasporogenesis and megagametogenesis [1], [2]. During megasporogenesis the Megaspore Mother Cell (MMC) undergoes meiosis and produces four haploid megaspores. Three of the megaspores are destined to degenerate, and just one will persist and become a functional megaspore (FM). This event marks the FG1 stage of megagametophyte development. Subsequently, the FM undergoes three consecutive mitotic divisions (FG1–FG4) that lead to the formation of the mature embryo sac (FG5) [1], [2]. The embryo sac is surrounded by the two integuments and connected to the placenta through the funiculus.

Active cross-talk between the maternal tissues and the developing embryo sac has been revealed from the study of ovule sporophytic mutants that display defects in megagametogenesis (for a review see [3]), and very recently it has been shown that auxin and cytokinins play key roles in ovule pattern formation [4]. Auxin moves from the placental tissue to the ovule [5] but the expression of genes involved in the tryptophan-dependent pathway of auxin production, such as *TAA* (*TRYPTOPHAN AMINOTRANSFERASE OF ARABIDOPSIS*) and *YUC* (*YUCCA*), indicate that auxin is also synthesized in developing ovules. The TAA enzymes convert tryptophan into IPA (indole-3-pyruvate), whereas the YUCs catalyse the conversion of IPA to IAA (indole-3-acetic acid) [6].

According to the accepted and well-documented model, the directionality of intercellular auxin flux mainly depends on the polar localization of the PIN proteins [7]. In *Arabidopsis* there are eight *PIN* genes (*PIN1–PIN8*), all of which may have diverged from a single ancestral gene sequence [8], [9].

In accordance with its function, the *pin1* mutant is affected in auxin transport [10] and displays progressive defects in organ initiation and phyllotaxy, leading to a pin-shaped inflorescence meristem devoid of flowers [11], [12]. A mutant allele of *PIN1*, *pin1*-*5*, develops flowers having abnormal pistils and containing only a few ovules, some of which display defective embryo sacs [13], [4]. This observation suggests a pivotal role for auxin during the early phases of megagametogenesis. In mature embryo sacs, overexpression of the auxin-producing enzyme *YUC1* and down-regulation of auxin-dependent transcription factors (*ARFs*) alter embryo sac cell identity [14], demonstrating that auxin also play an important role in the later stages of megagametogenesis.

In this manuscript we explore the roles PIN1 and PIN3 in ovule development by analysing *pin1-5* and *pin3-4* mutants and by silencing the wild-type genes using the *DEFIFICENS Homologue 9* ovule-specific promoter [15]. Our data indicate that PIN1 is required for correct female gametophyte development and we therefore propose that auxin directly or indirectly controls female gametogenesis and coordinates its developmental progression.

## Materials and Methods

### Plant Materials

*DR5rev:GFP, PIN1:PIN1-GFP, PIN4:GUS*, *PIN6:GUS* and *PIN7:PIN7-GFP* seeds were supplied by J. Friml (VIB Department of Plant Systems Biology, Gent). M. Heisler (EMBL, Developmental Biology, Heidelberg) and Y. Zhao (University of California at San Diego) supplied *pDR5rev:3XVENUS-N7* and *YUCCA4:GUS* seeds, respectively. *PIN3:PIN3-GFP*, *pin1-5* and *TAA1:GFP* seeds were donated by E. Benkova. *pin3-4* seeds were obtained from the NASC stock centre. The *pin3-4* mutant allele was amplified with oligonucleotides Atp4038 and LBp61, and the wild-type allele was amplified with Atp4038 and Atp2595. *PIN2:PIN2-GFP* was generated by insertion of CATGFP into the *PIN2* coding sequence at position 1436 from ATG, and its expression was driven by a 1.3 kb promoter region upstream of the *PIN2* locus.

### Quantitative Reverse Transcription PCR

Expression analyses of *PIN1* and *PIN3* were performed using the iQ5 Multi Color real-time PCR detection system (Bio-Rad). *PIN1* and *PIN3* specific primers are listed in Table S1; normalisation was performed using *UBIQUITIN10* (*UBI10*), r*RNA18* and PROTEIN PHOSPHATASE 2A SUBUNIT A3 (*PP2A3)* as internal standards.

Transcript abundances were confirmed by two independent biological experiments and four technical repetitions. Moreover, *PIN1* was amplified with two different primer pairs.

### GUS Assays and Whole-mount Preparation

GUS staining was performed as reported by Vielle-Calzada and collaborators [16]. Developing ovules were cleared according to Yadegari et al. [17] and observed using a Zeiss Axiophot D1 microscope (http://www.zeiss.com) equipped with differential interface contrast (DIC) optics. Images were recorded with an Axiocam MRc5 camera (Zeiss) using the Axiovision program (4.1).

### Confocal Laser Scanning Microscopy (CLSM ) and Immunolocalization

Dissected carpels were mounted with glycerol 5% (v/v). CLSM analysis was performed using a Zeiss LSM510 Meta confocal microscope.

To detect the GFP signal a 488 nm wavelength laser was used for excitation and a BP 505–550 nm filter was applied for GFP emission. For FM® 4–64 FX dye application (Invitrogen) an additional BP 575–615 nm filter was used.

For ovule morphological analyses pistils were fixed as described by Braselton et al. (1996) [18]. Samples were excited using a 532 nm laser and emission was detected between 570 and 740 nm.

For the immune-detection experiment a monoclonal anti-PIN1 antibody (Nanotools GmbH) was used as the primary antibody. To detect the signal from the Alexa Fluor® 555 secondary antibody (Invitrogen) a 561 nm laser was used for excitation and a LP 575 nm filter was applied for fluorophore emission.

### Plasmid Construction and Arabidopsis Transformation

All constructs were verified by sequencing and used to transform wild-type (Col-0) plants using the ‘floral-dip’ method [19].

Sequences for the artificial-mRNA against *PIN1* (*amiPIN1*) and *PIN3* (*amiPIN1-3*) were constructed according to Schwab et al. [20]. Primers used for *amiPIN1* and *amiPIN1-3* were Atp1104-07 and Atp1124-27. The cloning vector pBGW [21] (http://gateway.psb.ugent.be/) was modified introducing the *pDEFH9* promoter [15] and a *T35S* terminator fragment (primers Atp1663 and Atp1664).

### *In situ* Hybridisation

*In situ* hybridisations with digoxigenin-labelled antisense RNA were performed as previously described [22]. A specific *PIN5* cDNA fragment was amplified with primers Atp1229 and Atp1240.

## Results

### PIN1 and PIN3 are Expressed in Sporophytic Tissues during Ovule Development

We have investigated the expression of the eight members of the Arabidopsis *PIN* gene family in ovules. *PIN2:PIN2-GFP, PIN4:GUS, PIN6:GUS* and *PIN7:PIN7-GFP* transgenic plants were analysed and revealed that *PIN* genes *2*, *4*, *6* and *7* are not expressed in developing ovules (Figure S1). *PIN5* also fails to be expressed in developing ovules, as shown by the *in situ* hybridisation performed with a *PIN5* antisense specific probe (Figure S1), and it has been demonstrated recently that *PIN8* is expressed exclusively in pollen grains [23].

**Figure 1:**
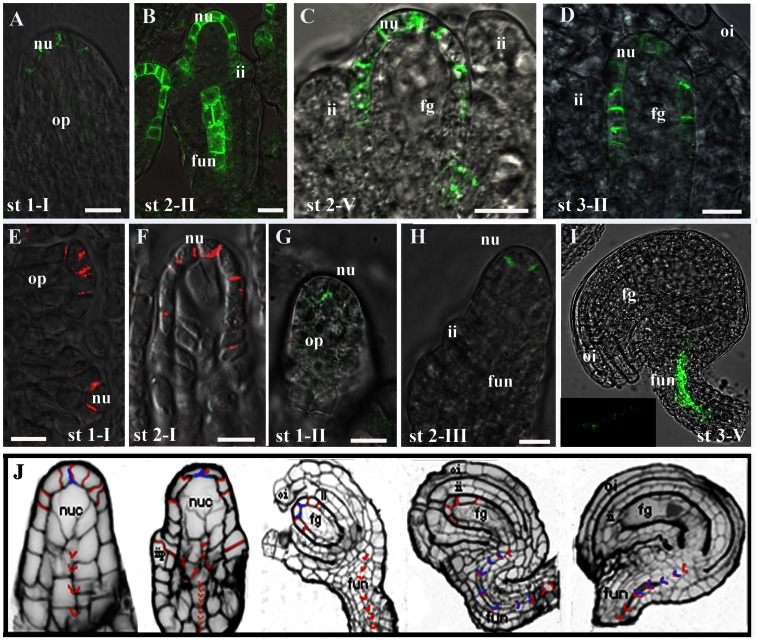
*PIN1* and *PIN3* expression in ovules. **A–F,** ***PIN1*** **; G–I** ***PIN3*** **A–D** **** CLSM analysis on *PIN1:PIN1-GFP* lines.At stage 1-I PIN1 is basal polar localized in the nucellar cells shortly after the formation of the ovule primordia (**A**). At stage 2–II, PIN1 is expressed in the nucellus and in the inner integument primordia (**B**). Closer view of a nucellus (ovule stage 2–V) (**C**). In developing ovules at stage 3-II, PIN1 is still basal polar localized (**D**). (**E–F**) PIN1 immuno-localisation experiments with an anti-PIN1 antibody. PIN1 is already detectable in the ovule primordium. (**E**) ovule primordia, stage 1–I; (**F**) ovule at stage 2–I. (**G–I**) CLSM analysis of *PIN3:PIN3-GFP*. PIN3 protein is expressed in the nucellus since stage 1–II (**G**) and its expression persists until stage 3–V (**I**). PIN3 is also expressed in the funiculus vasculature throughout ovule development, in the insert a closer view of the funiculus vasculature (**I**). In (**J)** a summary of PIN1 and PIN3 localisation in developing ovules. Ovule developmental stages are labelled following the convention of Schneitz and co-workers [1]. fg, female gametophyte; ii, inner integument; oi, outer integument; fun, funiculus; nu, nucellus. Scale bars: 20 µm.

With regard to PIN1, we performed a detailed PIN1 expression analysis from the earliest stages of ovule primordium development (stage 1–I) [1]. Analyses of transgenic plants containing the *PIN1:PIN1-GFP* construct demonstrate that PIN1 protein is localized at the lateral-apical membranes of diploid cells in the nucellus of developing ovule primordia (Figure 1A). From stage 1–II to stage 3–II, PIN1 was also detected in the inner integument and in the funiculus (Figures 1B). After stage 3–II the nucellus cells degenerate and PIN1 is restricted to the chalaza (Figure 1C and 1D). These data were confirmed by immuno-localisation experiments using specific antibodies against PIN1 (Figures 1E and 1F) [12]. PIN1 is expressed in the funiculus during all stages of ovule development.

Analysis of *PIN3:PIN3-GFP* plants [24] showed PIN3 to be present in the cells of the developing nucellus shortly after ovule primordium appearance (stage 1–II, Figure 1G). The PIN3-GFP chimeric protein persists and is detectable until stage 3–VI; PIN3 is also present in the funiculus starting from stage 2–III until late stages of ovule development (Figures 1H and I).

### Auxin Synthesis and Accumulation during Ovule Development

Since it is very difficult to measure cellular auxin levels, IAA distribution has often been inferred from the analysis of PIN protein orientation across tissues coupled to patterns of auxin response revealed using synthetic auxin-responsive reporter lines [25].

**Figure 2:**
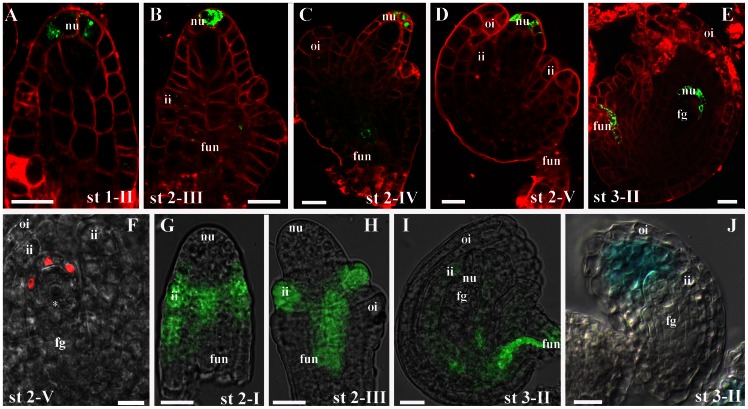
The Auxin synthesis and accumulation in wild type ovules. Wild-type *DR5rev:GFP* (**A–E**) and *DR5rev:3XVENUS-N7* (**F**) ovules were analysed at different developmental stages. The cytoplasmatic GFP signal is first detected in the nucellus of the ovule primordium from stage 1–II (**A**) until stage 3–III (**D**). *DR5rev:GFP* signal is also detected in the forming ovule vasculature (asterisks in **C** and **E)**. (**F**) the florescent signal confirms that the auxin response is only detected in the maternal nucellar cells, the YFP is fused to a nuclear localization sequence. In **A**–**D** cell membranes were stained with FM® 4–64 FX. Wild-type *TAA1:GFP* expression pattern in developing ovules at stages 2–I (**G**), 2–III (**H**), and 3–II (**I**). (**J**) YUC4:GUS is expressed since stage 3–II in the inner integument cells. The *YUC4* promoter is active in the inner integument in the cells close to the micropyle starting from stage 3–II. fg, female gametophyte; ii, inner integument; oi, outer integument; fun, funiculus; nu, nucellus Scale bars: 20 µm.

To investigate auxin accumulation in developing ovules we used plants carrying the auxin-responsive reporter *DR5rev:GFP* [26] which provides a convenient tool to study auxin distribution in developing organs [5], [27]. In the wild-type background the GFP signal appears in the epidermal cell layer of the nucellus of the ovule primordium starting from stage 1–II (Figure 2A). From stage 2–IV [1], [2] the auxin response was also detected in the pro-vascular cells of the funiculus (Figure 2C), and was further confirmed in the sporophytic nucellus of stage 2–V ovules by analysis of *DR5rev:VENUS-N7* transgenic plants (Figure 2F) [28] in which the *DR5rev* synthetic promoter drives three tandem copies of VENUS (a rapidly folding YFP variant) fused to a nuclear localization sequence. These observations clearly show that the auxin response is restricted to the micropylar pole of the nucellus until FG3 (stage 3–II), however auxin accumulation inside the female gametophyte could not be detected. This pattern is maintained until the nucellus progressively degenerates and is substituted by the endothelium (Figure S2).

To verify whether auxin is locally synthesized we have examined the expression of the genes *TAA1* and *YUC4* which are involved in the Trp-dependent auxin biosynthetic pathway [6], [29], taking advantage of transgenic lines expressing reporter genes (*GFP/GUS*) under the control of the *TAA1* or *YUC4* promoters. The *TAA1* promoter is active from stage 2–I (Figure 2G), and at stage 2–III the GFP signal is also detected in inner integument primordia and in the funiculus (Figure 2H). At stage 3–II the *TAA1* promoter drives GFP expression in those cells surrounding the female gametophyte (FG3) and in the funiculus (Figure 2I). The *YUC4* promoter [29] starts to be active at stage 3–II when the female gametophyte is at stage FG3 (Figure 2J) and remains active till stage 3–VI (Figure S3). Taken together, the data regarding auxin biosynthesis along with that on the localisation of the PIN1 and PIN3 proteins are consistent with auxin accumulation in the nucellus tissues.

### PIN1 is Required for Megametogenesis Progression

To unravel the importance of PIN1 and PIN3 in ovule development, we adopted a genetic approach.

In an initial step, we analysed the seed set of 10 *PIN1pin1* heterozygous plants (Gabi-KAT line GK_051A10). All ovules in these plants, half of which contain a *pin1* female gametophyte, were successfully fertilized by wild-type pollen in reciprocal crosses. This excludes the possibility that the *pin1* mutation has gametophytic effects.

**Figure 3:**
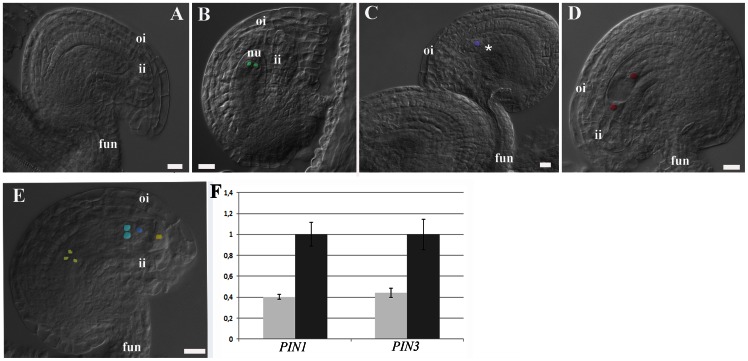
*PIN1* down regulation affects ovule development. (**A** and **B**) *pin1-5* ovules with defective female gametophyte. The female gametophytes arrest in development at FG1. (**C** and **D**) ovules of the PIN1 down-regulation (*pDEFH9:amiPIN1)* plants show female gametophyte defects. Female gametophytes are blocked at stages FG1 and FG3. (**E**) mature wild-type ovules, with FG7 female gametophyte. (**F**) Real-time PCR to quantify *PIN1* and *PIN3* expression levels in transgenic plants expressing the artificial microRNA against *PIN1* and *PIN3* (light grey transgenic plants showing ovule abortion, dark grey sibling wild-type plants with normal seed set). Expression of the artificial microRNA is under the control of the ovule specific *pDEFH9* promoter. Scale bars: 20 µm nu, nucellus; fun, funiculus; ii, inner integument; oi, outer integument; fg, female gametophyte.

*pin1* homozygous plants develop a pin-shaped inflorescence meristem devoid of flowers [11], [12]. Thus we analysed the *pin1-5* allele to elucidate the sporophytic role of PIN1 during ovule development [13], [30]. *pin1*-*5* plants develop flowers containing very few ovules compared to wild-type. In an analysis of 183 ovules, Bencivenga and co-workers [4] reported that 10% of them develop as finger-like structures, whilst 70% showed defects in megagametogenesis since most of the embryo sacs arrested in FG1 or FG3, corresponding to ovule developmental stages 3–I and 3–III (Figures 3A and 3B, Table S2A) [1], [4].

The phenotype described in the *pin1-5* mutant could be due to the additive effect of reduced auxin flux in the developing pistil and reduced auxin flux during ovule development. To uncouple the two possible causes of the observed phenotype in *pin1-5* female gametophytes, we silenced *PIN1* with an artificial micro-RNA (*amiPIN1*; [20]) under the control of the ovule-specific promoter *pDEFH9* [15]. *DEFH9* is an *Antirrhinum majus* ovule-specific gene and its promoter is active in *Arabidopsis* ovules from stage 1–II (Figure S4). Among 50 *DR5rev:GFP* T_1_ plants containing the *pDEFH9:amiPIN1* construct, 18 were characterised by an abnormal seed set with a significant percentage of their ovules (from 26% to 72%) unable to complete proper development (five siliques per plant were manually dissected). Analysis of the T_2_ population (T_1_ pollen was used to pollinate wild-type plants) using BASTA selection showed that 15 out of the 18 plants analysed had only one T-DNA copy (or multiple T-DNA copies in linkage segregating as a single locus - data not shown). Optical microscopic analysis showed that ovules from these 15 transgenic plants were blocked at either the FG1 stage (10 lines) or at the FG3 stage (5 lines) (Figures 3C and 3D) resulting in a high percentage of ovule abortions (Table S2B). These data in conjunction with the observation that *PIN1pin1* heterozygous plants have normal seed sets clearly suggest that the observed gametophytic defects in *pDEFH9:amiPIN1* lines are of sporophytic origin. This is also in accord with the nature of the *pin1-5* ovule phenotype.

To further support the contention that ovule abortion was due to *PIN1* down-regulation, we performed real-time PCR analysis on the carpels of the T_2_ segregating plants using either *UBIQUITIN10* or *18S RNA* as reference genes (Figure S5). This analysis showed that *PIN1* transcript levels were reduced by 2 to 5 fold in those p*DEFH9:amiPIN1* plants producing defective female gametophytes (Figures 3C and D) in comparison to sibling wild-type plants. Interestingly, analysis of the *DR5rev:GFP* reporter in the *PIN1* silenced lines showed that those ovules that were unable to complete megagametogenesis maintained an auxin maximum at the distal edge of the blocked embryo sacs. Such an auxin response is not observed in wild-type sister FG5 ovules (Figure S6). The persistence of auxin activity was associated with the block of gametogenesis, however the sporophytic tissues of these ovules proceeded with development and reached maturity. These data suggest that PIN1 is involved in auxin flux towards the female gametophyte which is required for gametophyte progression.

### PIN3 Function is not Required for Ovule Development

PIN3 is also expressed during ovule development as demonstrated in *pPIN3:PIN3-GFP* plants. To elucidate a potential role for PIN3 in ovule development we have analysed *pin3-4* homozygous mutant plants. These plants do not show any ovule defects and are fully fertile. To assess whether PIN3 plays a role in ovule development in *pin1* down-regulated plants, we transformed wild-type plants with a micro RNA against *PIN3* and *PIN1* under the control of the *DEFH9* promoter (*pDEFH9:amiPIN1-3*). We selected 9 transgenic plants characterised by the presence of ovule defects (Table S2C). In these plants, both *PIN1* and *PIN3* were down-regulated and the ovule arrest in, FG1 or FG3 (Figure 3F) *pin1-5* and *pDEFH9:amiPIN1* plants. The percentage of ovule abortions (values varied greatly in different plants ranging from 45% to 70%) is not significantly different compared to the *PIN1* silenced plants, indicating that PIN1 is the major player in female gametophyte progression.

## Discussion

### *PIN1* is Expressed in Ovule Sporophytic Cells and Promotes Female Gametophyte Development

**Figure 4:**
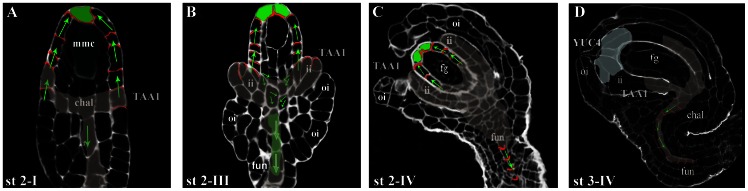
Model of auxin flux and production during ovule development. The diagram summarises the auxin response (in green), auxin flux (red arrows) and auxin synthesis (grey) in developing ovules from stage 2–I (**A**) to stage 3–IV (**D**). At stage 2–I (**A**) the megaspore mother cell (MMC) is recognisable. At this stage the auxin response (*DR5rev::GFP)* is restricted to a few cells of the nucellus; PIN1 is also detected in the nucellar cells and has is a basal polar localization. At this stage *pTAA1* drives reporter expression in the ovule chalaza region and in the funiculus. At stage 2–III (**B**) PIN1 is detected in the inner integument primordia and in the funiculus, comcometeley with the auxin response. At stage 2–IV (**C**) the *TAA1* promoter reduces its expression in the inner integument but at the tip of the inner integument *YUC4* begins to be expressed. At this stage PIN1 and the auxin response are restricted to the funiculus since the nucellus is degenerating. The position of PIN1 in the nucellus cells strongly indicates its importance in auxin exit from these cells. The exit of auxin stimulates megagametogenesis as shown by *PIN1* silencing. fg, female gametophyte; ii, inner integument; oi, outer integument; fun, funiculus; chal, chalaza.

Based on our data on local auxin synthesis, PIN localization and auxin response patterns we propose a dynamic model describing auxin flux during ovule development (Figure 4A–D).

Shortly after ovule primordium formation, PIN1 is detectable in the nucellus cells. PIN1 membrane localisation clearly indicates the direction of auxin flux in the nucellus (Figure 4A–C), leading to auxin accumulation in specific nucellus cells (Figure 4A–C).

The location of PIN1 in the nucellus cells (Figure 4A–C) suggests that either auxin could be transported into the forming embryo sacs and regulate gametophytic progression, or alternatively it might regulate the expression of unidentified factor/s in the nucellus that directly or indirectly promote megagametogenesis. Identification of the target genes regulated by the auxin response factors required for female gametophyte development will contribute to discriminate between the two possibilities.

The accumulation of auxin in the female gametophyte was described previously by Pagnussat and co-workers [14], however we did not detect an auxin responce in the MMC or in female gametophytes (Figure 4A–D) despite the long half-life of the reporter protein GFP [31]. Nevertheless, our observations clearly demonstrate that sporophytic PIN1 expression in the ovule (Figure 4A–C) is needed for the early phases of embryo sac formation since *PIN1* down-regulation compromises embryo sac development. The importance of PIN1 in early and later stages of female gametophytic development has been proposed previously: the disruption of cytokinin signalling in the ovule’s sporophytic tissue results in the down-regulation of PIN1 and the lack of embryo sac formation [4]. It has been shown that *PIN1* expression is under the control of SPOROCYTLESS/NOZZLE (SPL/NZZ) [4]. Interestingly, while *spl* ovules do not develop the MMC [32], the majority of ovules in both *pin1-5* and *pDEFH9:amiPIN1* plants are able to [1]. Thus whilst PIN1 plays a key role in promoting megagametogenesis, this observation suggests that MMC formation does not require PIN1 expression.

Although PIN1 seems to be the only member of the PIN protein family to play an important role in ovule development, we cannot at present exclude the possibility that other auxin transporters are also required to dynamically coordinate auxin flux, such as the ABC transporters [33].

### Auxin Might Play a Major Function in Haploid/diploid Generation Cross-talk

The importance of dynamic gradients is strengthened by the tight temporal and spatial regulation of auxin biosynthesis in ovules (Figure 4A–D). For instance, the expression of both *TAA1* and *YUC4* genes is restricted to a small group of cells during ovule development. *TAA1* is expressed within the ovule primordium and at later developmental stages in the chalaza region of stage 2–IV ovules and in the developing inner integuments (Figure 4A–D). *YUC4* is expressed at later stages than *TAA1*, starting at FG2 and being restricted to the tip of developing inner integument (Figure 4A–D).

Several genetic screenings (for a review see [34]) have been performed to identify genes involved in megagametophyte development. To date all the gametophytic mutants described develop ovule sporophytic tissues correctly. In contrast, several ovule mutants with sporophytic defects show anomalies in gametophyte development. Therefore, it has been proposed that a hierarchy exists in the communication between ovule sporophytic tissues and the gametophytic haploid generation, and that greater importance is attributed to the sporophytic maternal tissues.

We propose that auxin (directly or indirectly) might have an important function in the communication between generations.

In conclusion, we propose that auxin (directly or indirectly) has an important function in the communication between generations. Previous work has shown that auxin is involved in several aspects of ovule development such as ovule primordium formation [35] and the determination of embryo sac cell identity [14]. The results presented in the current work highlight the involvement of auxin flux modulation in the progression of megagametogenesis.

## Supporting Information

Figure S1 PIN2, *PIN4, PIN5, PIN6* and PIN7 are not expressed in developing ovules. **(A)** a *PIN2:PIN2-GFP* ovule at stage 2–IV and a primary root (B); **(C)** a *PIN4:GUS* ovule at stage 2–III and a primary root (D)*;* **(E)** *in situ* hybridisation to developing ovules (stage 2–III) using an antisense *PIN5* probe, and to developing stamens and carpel leaves (F). **(G)** a *PIN6:GUS* developing ovule (stage 2–IV) and a primary root (H). **(I)** a *PIN7:PIN7-GFP* ovule (stage 2–III) and a primary root (J). The absence of signals in ovules compared to that seen in other tissues indicates that the genes under test are not expressed in ovules.fun, funiculus; ii, inner integument; oi, outer integument; ov, ovule; pi, pistil; pl, placenta.
(TIF)

Figure S2 Ovule development and the auxin response. (**A,C**) Confocal analysis of wild-type ovules, (**B,D**) *DR5rev:3XVENUS-N7* ovules at the corresponding stages. In C and D the endothelium begins to develop (see arrow) whereas the nucellar cells degenerate and accordingly the fluorescent nuclear auxin response is no longer detectable, though the auxin response is still visible in the funiculus (asterisk). Scale bars: 20 µm fg, female gametophyte; ii, inner integument; oi, outer integument; fun, funiculus; nu, nucellus; end, endothelium.
(TIF)

Figure S3 *YUC4* is expressed in developing ovules. The *YUC4* promoter (*pYUC4* ***)*** does not drive reporter gene expression (GUS) in developing ovules between stages 1–I (**A**) and 3–I (**C**). Reporter gene activity begins to be detected in ovules at stage 3–II (**D**) and it is maintained until stage 3–V (**E**). fg, female gametophyte; ii, inner integument; oi, outer integument; fun, funiculus Scale bars: 20 µm.
(TIF)

Figure S4 *pDEH9* is an ovule-specific promoter also in *Arabidopsis thaliana.* The *Antirrhinum majus DEF9* promoter (*pDEH9* ***)*** drives reporter gene expression (GUS) only in developing ovules, the promoter being active from stage II **(A)** to stage 3–V **(E).** fg, female gametophyte; ii, inner integument; oi, outer integument; fun, funiculus; nu, nucellus.
(TIF)

Figure S5 *PIN1* expression in *pDEFH9:amiPIN1* flowers. Real-time PCR to evaluate *PIN1* expression in *pDEFH9:amiPIN1* flowers. Two pairs of *PIN1* specific primers were employed (orange and green).
(TIF)

Figure S6 The auxin response in normal and mutated ovules of *pDEFH9:amiPIN1* plants. (A) The *DR5rev:GFP* promoter is active in mutated ovules unable to complete megagametogenesis. fg, female gametophyte; ii, inner integument; oi, outer integument; fun, funiculus; nu, nucellus Scale bars: 20 µm.
(TIF)

Table S1 A Analysis of *pin1-5* developing ovules. 20 pistils collected from 4 independent plants have been analysed. B and C Analysis of T2 *pDEFH9:amiPIN1* and *pDEFH9:amiPIN1-3* plants. The total number of ovules and the percentage of ovule abortion are reported. 5 siliques per individual were observed, and 15 independent lines have been analysed. Aberrant embryo sac arrest at FG1 or FG3 stages (Figure 3). D Analysis of *pin3-4* developing ovules. 10 pistils collected from 4 independent plants have been analysed.
(DOCX)

Table S2 Sequences of oligonucleotide primers used in this work.
(DOCX)
