# Supplementary figures and images for: Maternal Control of PIN1 Is Required for Female Gametophyte Development in Arabidopsis
Source: PLoS One. 2013 Jun 17;8(6):e66148. doi: 10.1371/journal.pone.0066148 (PMC3684594; doi:10.1371/journal.pone.0066148)

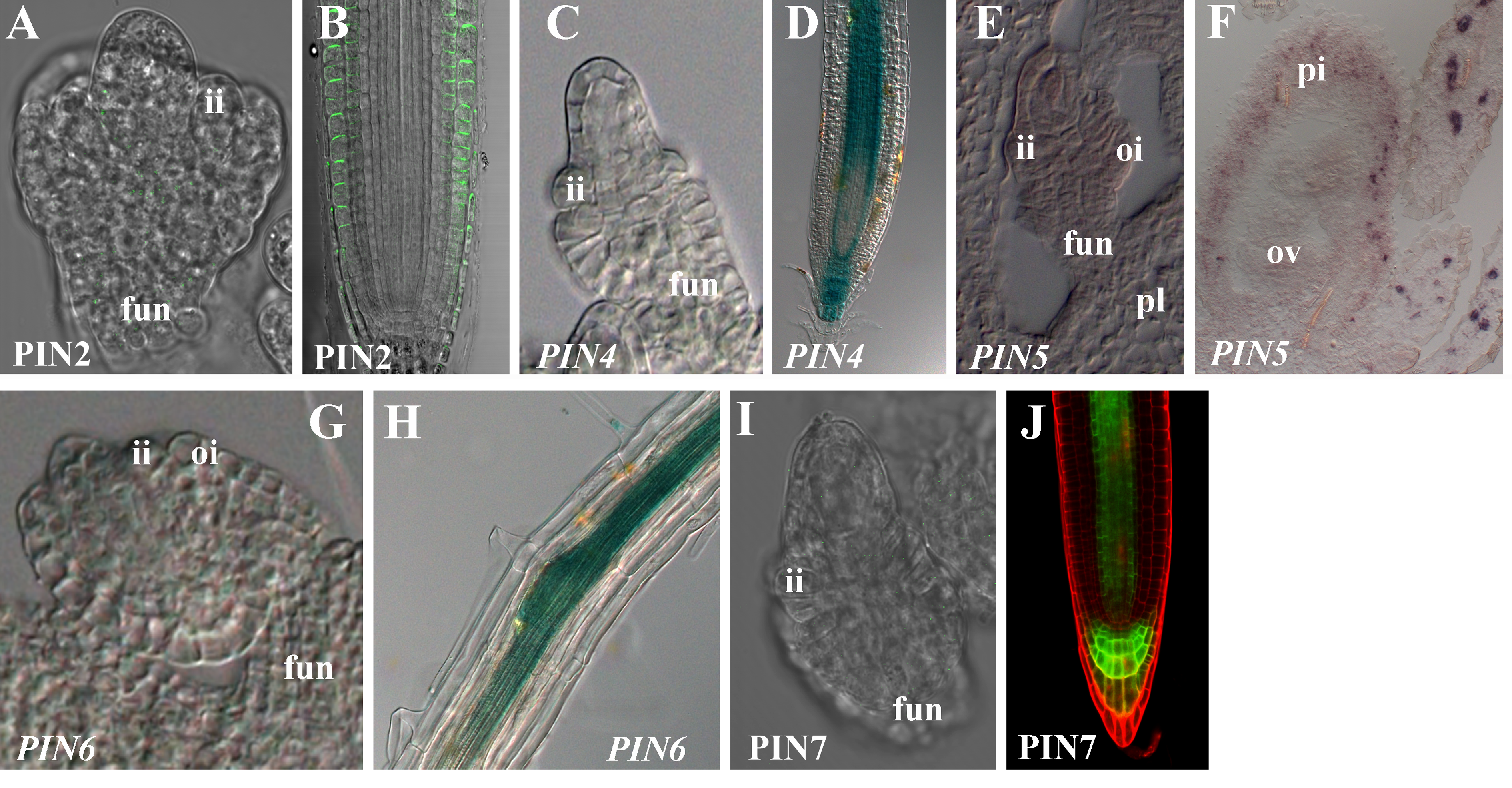

Supplement: Figure S1 — PIN2, PIN4, PIN5, PIN6 and PIN7 are not expressed in developing ovules. (A) a PIN2:PIN2-GFP ovule at stage 2–IV and a primary root (B); (C) a PIN4:GUS ovule at stage 2–III and a primary root (D); (E) in situ hybridisation to developing ovules (stage 2–III) using an antisense PIN5 probe, and to developing stamens and carpel leaves (F). (G) a PIN6:GUS developing ovule (stage 2–IV) and a primary root (H). (I) a PIN7:PIN7-GFP ovule (stage 2–III) and a primary root (J). The absence of signals in ovules compared to that seen in other tissues indicates that the genes under test are not expressed in ovules.fun, funiculus; ii, inner integument; oi, outer integument; ov, ovule; pi, pistil; pl, placenta. (TIF) [file pone.0066148.s001.tif]

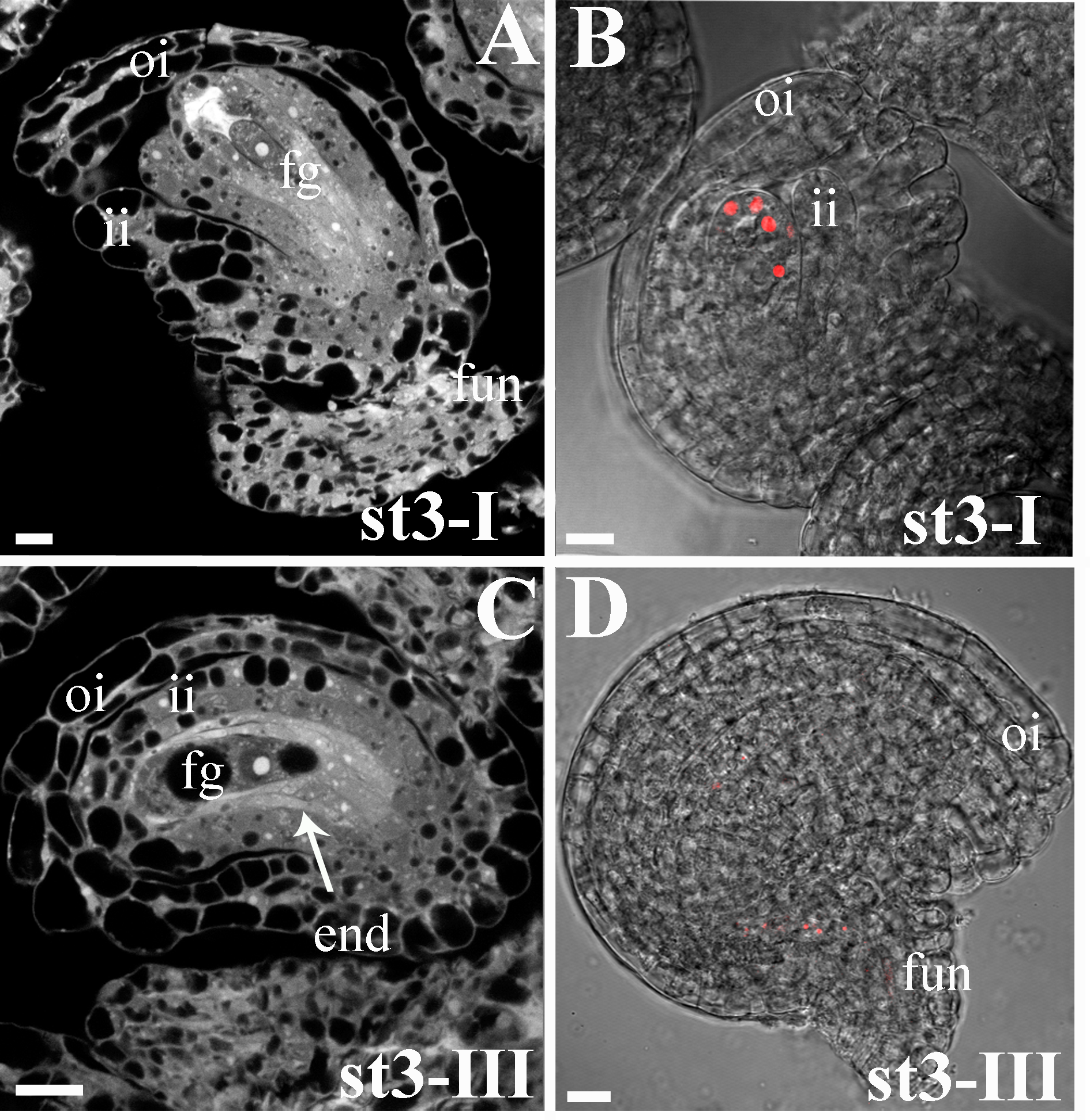

Supplement: Figure S2 — Ovule development and the auxin response. (A,C) Confocal analysis of wild-type ovules, (B,D) DR5rev:3XVENUS-N7 ovules at the corresponding stages. In C and D the endothelium begins to develop (see arrow) whereas the nucellar cells degenerate and accordingly the fluorescent nuclear auxin response is no longer detectable, though the auxin response is still visible in the funiculus (asterisk). Scale bars: 20 µm fg, female gametophyte; ii, inner integument; oi, outer integument; fun, funiculus; nu, nucellus; end, endothelium. (TIF) [file pone.0066148.s002.tif]

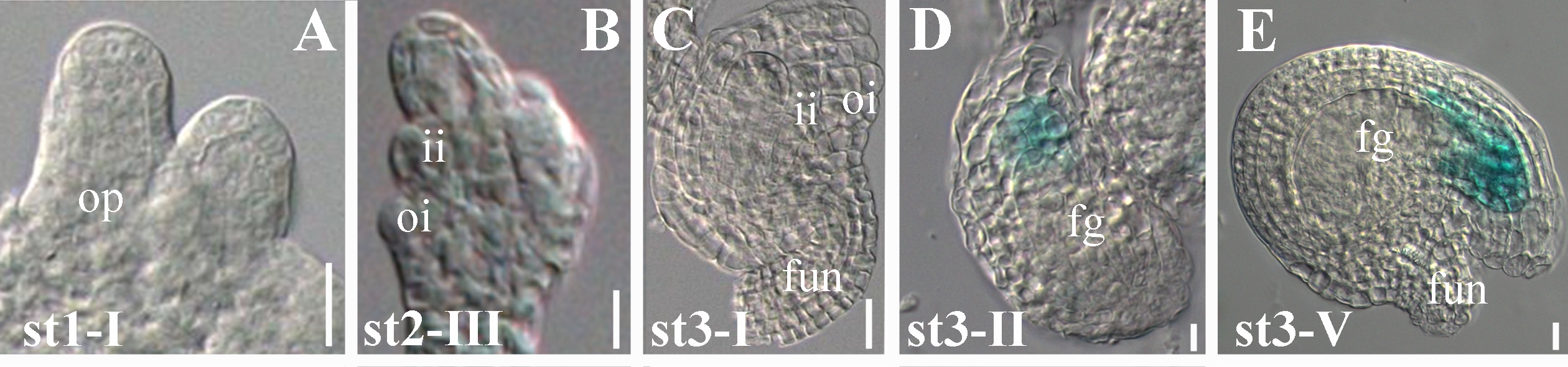

Supplement: Figure S3 — YUC4 is expressed in developing ovules. The YUC4 promoter (pYUC4 ) does not drive reporter gene expression (GUS) in developing ovules between stages 1–I (A) and 3–I (C). Reporter gene activity begins to be detected in ovules at stage 3–II (D) and it is maintained until stage 3–V (E). fg, female gametophyte; ii, inner integument; oi, outer integument; fun, funiculus Scale bars: 20 µm. (TIF) [file pone.0066148.s003.tif]

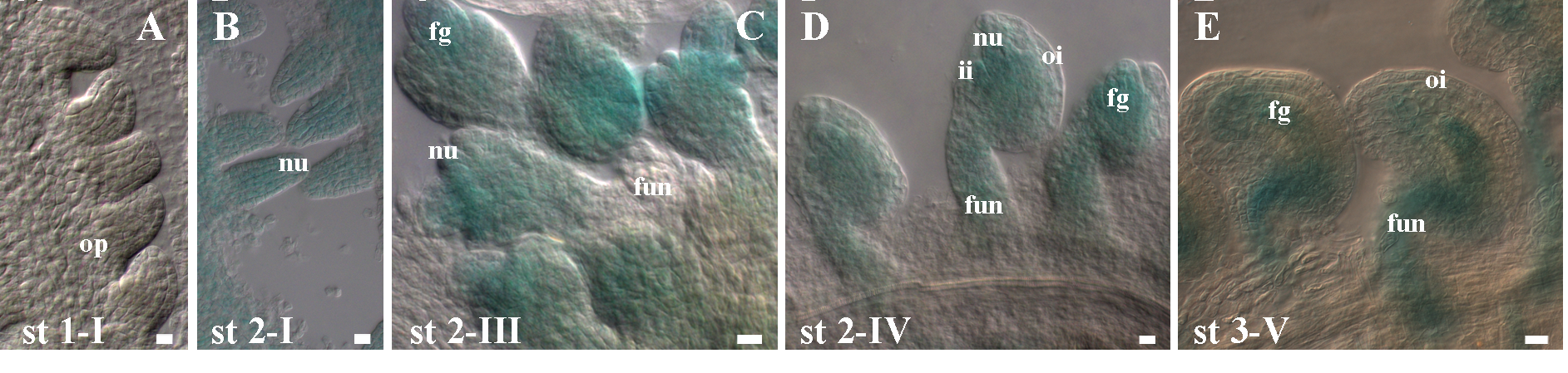

Supplement: Figure S4 — pDEH9 is an ovule-specific promoter also in Arabidopsis thaliana. The Antirrhinum majus DEF9 promoter (pDEH9 ) drives reporter gene expression (GUS) only in developing ovules, the promoter being active from stage II (A) to stage 3–V (E). fg, female gametophyte; ii, inner integument; oi, outer integument; fun, funiculus; nu, nucellus. (TIF) [file pone.0066148.s004.tif]

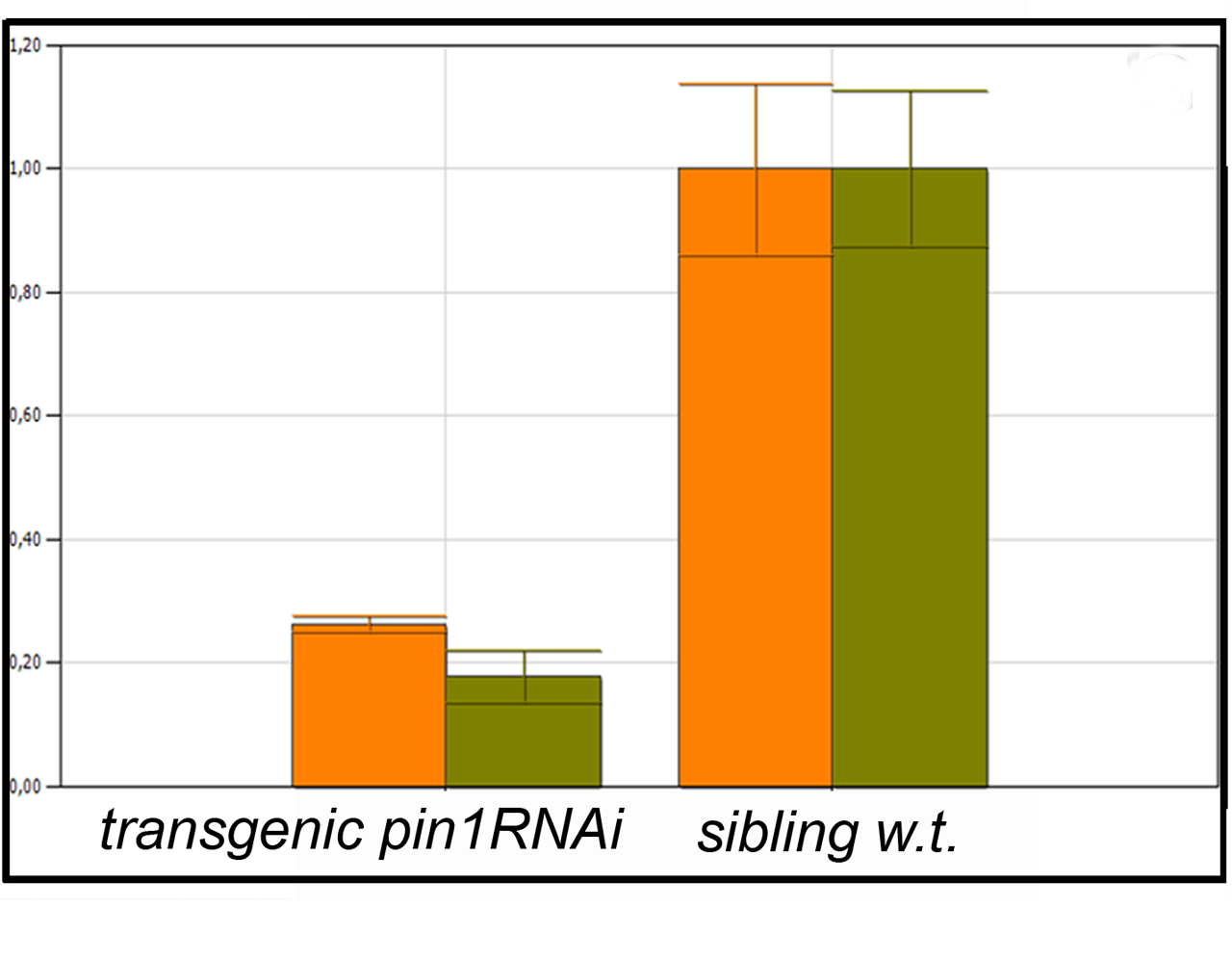

Supplement: Figure S5 — PIN1 expression in pDEFH9:amiPIN1 flowers. Real-time PCR to evaluate PIN1 expression in pDEFH9:amiPIN1 flowers. Two pairs of PIN1 specific primers were employed (orange and green). (TIF) [file pone.0066148.s005.tif]

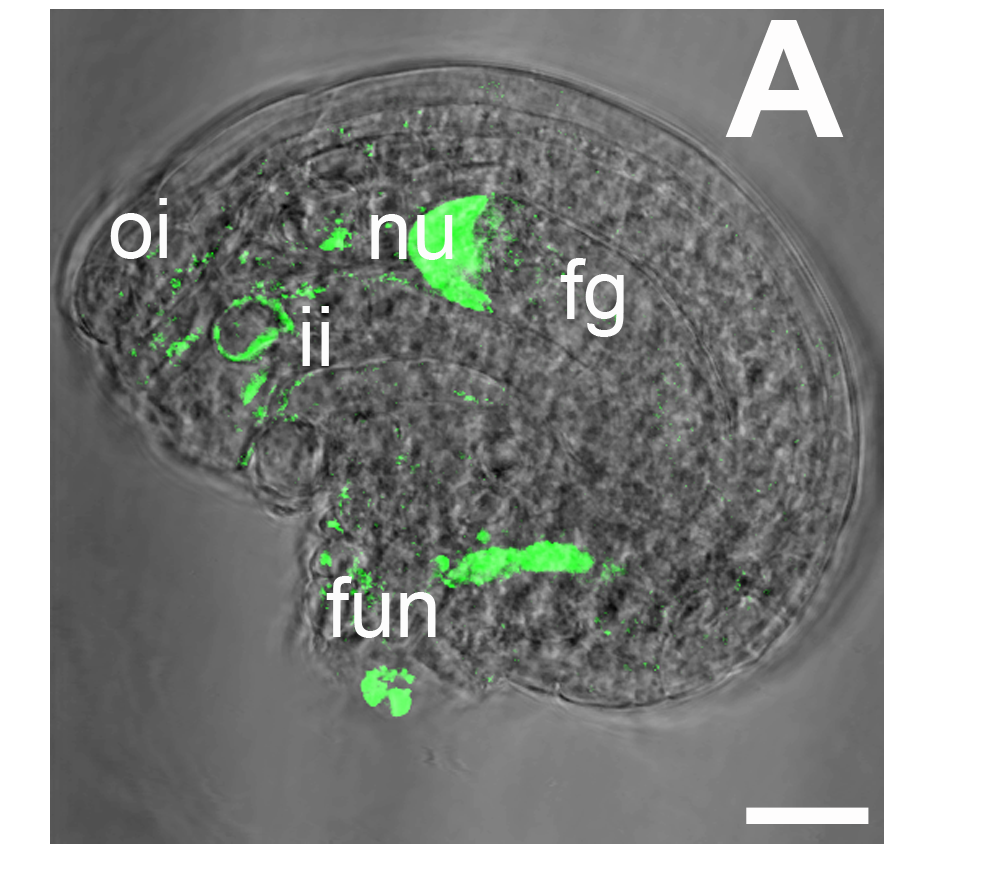

Supplement: Figure S6 — The auxin response in normal and mutated ovules of pDEFH9:amiPIN1 plants. (A) The DR5rev:GFP promoter is active in mutated ovules unable to complete megagametogenesis. fg, female gametophyte; ii, inner integument; oi, outer integument; fun, funiculus; nu, nucellus Scale bars: 20 µm. (TIF) [file pone.0066148.s006.tif]
