# Supplementary material for: Maternal Control of PIN1 Is Required for Female Gametophyte Development in Arabidopsis
Source: PLoS One. 2013 Jun 17;8(6):e66148. doi: 10.1371/journal.pone.0066148 (PMC3684594; doi:10.1371/journal.pone.0066148)
Supplement: Table S1 — A Analysis of pin1-5 developing ovules. 20 pistils collected from 4 independent plants have been analysed. B and C Analysis of T2 pDEFH9:amiPIN1 and pDEFH9:amiPIN1-3 plants. The total number of ovules and the percentage of ovule abortion are reported. 5 siliques per individual were observed, and 15 independent lines have been analysed. Aberrant embryo sac arrest at FG1 or FG3 stages (Figure 3). D Analysis of pin3-4 developing ovules. 10 pistils collected from 4 independent plants have been analysed. (DOCX) [file pone.0066148.s007.docx]

**Table S1**

**Sequences of primers used in this work**

| At2208 | CACCAAGGTCTCAAGGCTTATCTGCG | PIN1 interference |
| --- | --- | --- |
| At2009 | TCCGTCTTGTCTTTTCCCACC | PIN1 interference |
| At590 | CTCAGAATTCGTTGGGTATGTTCTCACTTTC | *STK* promoter |
| At591 | GTCACTCGAGTCCCATCCTTCATTTTAAACAT | *STK* promoter |
| At1507 | TCTGACGTCAGGCGTTTTTGTTGGGTATGTTCTCAC | *STK* promoter |
| At1508 | TCTGACGTCAGGCATCCTTCATTTTAAACATC | *STK* promoter |
| At1663 | CGAGCTCGCGGCCATGCTAGAGTCCGC | 35STer |
| At1664 | CGAGCTCGAGGTCACTGGATTTTGGTTTTAGG | 35STer |
| At2719 | TTGTGGAGATGTTTACAAGG | PIN5 in situ probe |
| At2720 | TGTTGTCTACTTGTACGTCG | PIN5 in situ probe |
| R-At331 | TGACGGAGAATTAGGGTTCG | real time rRNA18 |
| R-At332 | CCTCCAATGGATCCTCGTTA | real time rRNA18 |
| R-At274 | CTTGGCTTGTAATGTTGGCATCAG | Real time PIN3 |
| R-At273 | GAGGGAGAAGGAAGAAAGGGAAAC | Real time PIN3 |
| R-At147 | CTGTTCACGGAACCCAATTC | Ubiquitin real time |
| R-At148 | GGAAAAAGGTCTGACCGACA | Ubiquitin real time |
| R-At2398 | CGACACTCCCCAACACTCTAG | Real time PIN1 |
| R-At2399 | AGCTTAGCTCCACGGTACTC | Real time PIN1 |
| R-At2404 | AGAAGCAGCTTTAGAGAATC | Real time PIN1 |
| R-At2405 | AAGAGGAAACACGAATGTTC | Real time PIN1 |
| R-At857 | TATCGGATGACGATTCTTCGTGCAG | Real time PPP2 |
| R-At858 | GCTTGGTCGACTATCGGAATGAGAG | Real time PPP2 |
| At3614 | GACGTTAATACGAATCAGCAG | PIN3 in situ probe |
| At3615 | GATGAGCTACAGCTTTGGTCTC | PIN3 in situ probe |
| At1104 | GATAACGTGGTAGAAGTCCCGCGTCTCTCTTTTGTATTCC | Artificial µRNA vs PIN1 |
| At1105 | GACGCGGGACTTCTACCACGTTATCAAAGAGAATCAATGA | Artificial µRNA vs PIN1 |
| At1106 | GACGAGGGACTTCTAGCACGTTTTCACAGGTCGTGATATG | Artificial µRNA vs PIN1 |
| At1107 | GAAAACGTGCTAGAAGTCCCTCGTCTACATATATATTCCT | Artificial µRNA vs PIN1 |
| At1124 | GATGCAAACACAAACGGTACGATTCTCTCTTTTGTATTCC | Artificial µRNA vs PIN1 &3 |
| At1125 | GAATCGTACCGTTTGTGTTTGCATCAAAGAGAATCAATGA | Artificial µRNA vs PIN1 &3 |
| At1126 | GAATAGTACCGTTTGAGTTTGCTTCACAGGTCGTGATATG | Artificial µRNA vs PIN1 &3 |
| At1127 | GAAGCAAACTCAAACGGTACTATTCTACATATATATTCCT | Artificial µRNA vs PIN1 &3 |

Atp173 5’-GGTCCACAACCTGGATGTGTTCG-3’

Atp582 5’GGGGACAAGTTTGTACAAAAAAGCAGGCTcatgtgtgttcatgtgaaaagc-3’

Atp583 5’GGGGACCACTTTGTACAAGAAAGCTGGGTttctattgccacttactctcac-3’

Atp1550 5’GGGGACCACTTTGTACAAGAAAGCTGGGTCtagctctacatgagaaacccac-3’

Atp1247 5’-CCCATTTGGACGTGAATGTAGACAC-3’Atp13075'-CATGTTTCTCGGGTAATCTGCG-3'

Atp1308 5'-GGCAATTCTTCTGGTGTATTC-3'

Atp1441 5’-tttagtcaagcaccatcatttacg-3’

Atp1544 5’-cacatctgcataagccttg-3’

Atp1981 5'-caaggcttatgcagatgtg-3'

Atp 2187 5'-GAGTTCTTGGTCTTGTCATC-3'

Atp 2188 5'-GATTTGGTAATAGCATATGTGTTG-3'
