# Supplementary material for: Maternal Control of PIN1 Is Required for Female Gametophyte Development in Arabidopsis
Source: PLoS One. 2013 Jun 17;8(6):e66148. doi: 10.1371/journal.pone.0066148 (PMC3684594; doi:10.1371/journal.pone.0066148)
Supplement: Table S2 — Sequences of oligonucleotide primers used in this work. (DOCX) [file pone.0066148.s008.docx]

**TABLE S1**

**A) Analysis of *pin1-5* developing ovules.**

Total number of ovules and aborted ovules per each carpel are reported.

| carpel | defect | Aborted ovules | Normal ovules | TOT |
| --- | --- | --- | --- | --- |
|  |  |  |  |  |
| **1** | FG1 | 7 | 1 | 8 |
| **2** | FG1 | 11 | 1 | 12 |
| **3** | FG1+FG3 | 11 | 3 | 14 |
| **4** | FG1 | 6 | 3 | 9 |
| **5** | FG1 | 2 | 5 | 7 |
| **6** | FG1 | 8 | 0 | 8 |
| **7** | FG1 | 9 | 0 | 9 |
| **8** | FG1 | 8 | 3 | 11 |
| **9** | FG3 | 6 | 5 | 11 |
| **10** | FG1 | 9 | 0 | 9 |
| **11** | FG1 | 5 | 0 | 5 |
| **12** | FG3 | 6 | 2 | 8 |
| **13** | FG1 | 6 | 2 | 8 |
| **14** | FG1 | 4 | 0 | 4 |
| **15** | FG3 | 8 | 4 | 12 |
| **16** | FG3 | 9 | 1 | 10 |
| **17** | FG1 | 8 | 0 | 8 |
| **18** | FG1 | 5 | 4 | 9 |
| **19** | FG1 | 9 | 3 | 12 |
| **20** | FG1 | 9 | 1 | 10 |
|  |  |  |  |  |

**B) Analysis of T2 *pDEFH9:amiPIN1* plants.**

Total number of ovules and percentage of ovule abortion are reported. 5 siliques per each individual have been observed, 15 independent lines have been observed. The aberrant embryo sacs arrest at FG1 or FG3 stage.

| Plant | defect | Aborted ovules | Normal ovules | TOT | Abortion % |
| --- | --- | --- | --- | --- | --- |
|  |  |  |  |  |  |
| **1** | FG1 | 73 | 208 | 281 | ***26%*** |
| **2** | FG1 | 117 | 133 | 250 | ***47%*** |
| **3** | FG1+FG3 | 143 | 22 | 165 | ***87%*** |
| **4** | FG1 | 65 | 79 | 144 | ***45%*** |
| **5** | FG1 | 60 | 104 | 164 | ***36.5%*** |
| **6** | FG1+3 | 63 | 76 | 139 | ***45%*** |
| **7** | FG1 | 107 | 93 | 200 | ***53.5%*** |
| **8** | FG1 | 146 | 57 | 203 | ***72%*** |
| **9** | FG3 | 114 | 145 | 259 | ***44%*** |
| **10** | FG1 | 140 | 227 | 367 | ***38%*** |
| **11** | FG1 | 109 | 117 | 227 | ***48%*** |
| **12** | FG3 | 99 | 85 | 184 | ***54%*** |
| **13** | FG1 | 111 | 99 | 200 | ***55%*** |
| **14** | FG1 | 130 | 61 | 191 | ***68.5%*** |
| **15** | FG3 | 113 | 144 | 257 | ***44%*** |

**C) Analysis of T1 *pDEFH9:amiPIN1-3* plants.**

Total number of ovules and percentage of ovule abortion are reported. 5 siliques per each individual have been observed, 9 independent lines have been observed. The aberrant embryo sacs arrest at FG1 or FG3 stage

| Plant | defect | Aborted ovules | Normal ovules | TOT | Abortion % |
| --- | --- | --- | --- | --- | --- |
|  |  |  |  |  |  |
| **1** | FG1 | 85 | 187 | 272 | ***31%*** |
| **2** | FG1 | 120 | 131 | 251 | ***48%*** |
| **3** | FG1+FG3 | 123 | 42 | 165 | ***69.6%*** |
| **4** | FG1 | 80 | 106 | 186 | ***43%*** |
| **5** | FG1 | 83 | 102 | 185 | ***45%*** |
| **6** | FG3 | 108 | 81 | 189 | ***57%*** |
| **7** | FG1 | 107 | 98 | 205 | ***52%*** |
| **8** | FG1 | 155 | 60 | 215 | ***70%*** |
| **9** | FG1 | 96 | 163 | 259 | ***37%*** |

**D) Analysis of *pin3-4* developing ovules.**

Total number of ovules and aborted ovules per each carpel are reported.

| carpel | Aborted ovules | Normal ovules | TOT |
| --- | --- | --- | --- |
|  |  |  |  |
| **1** | 2 | 49 | 51 |
| **2** | 3 | 55 | 58 |
| **3** | 2 | 55 | 57 |
| **4** | 2 | 48 | 50 |
| **5** | 2 | 5 | 59 |
| **6** | 2 | 49 | 51 |
| **7** | 3 | 54 | 57 |
| **8** | 2 | 54 | 56 |
| **9** | 2 | 55 | 57 |
| **10** | 4 | 50 | 54 |
